# An external evaluation of the Diarrhea Alleviation through Zinc and ORS Treatment (DAZT) program in Gujarat and Uttar Pradesh, India

**DOI:** 10.7189/jogh.05.020409

**Published:** 2015-12

**Authors:** Laura M Lamberti, Sunita Taneja, Sarmila Mazumder, Amnesty LeFevre, Robert E Black, Christa L Fischer Walker

**Affiliations:** 1 Johns Hopkins Bloomberg School of Public Health. Department of International Health, Baltimore, MD, USA; 2 Society for Applied Studies, Centre for Health Research and Development, New Delhi, India

## Abstract

**Background:**

To address inadequate coverage of oral rehydration salts (ORS) and zinc supplements for the treatment of diarrhea among children under–five, the Diarrhea Alleviation through Zinc and ORS Treatment (DAZT) program was carried out from 2011–2013 in Gujarat and from 2011–2014 in Uttar Pradesh (UP), India. The program focused on improving the diarrhea treatment practices of public and private sector providers.

**Methods:**

We conducted cross–sectional household surveys in program districts at baseline and endline and constructed state–specific logistic regression models with generalized estimating equations to assess changes in ORS and zinc treatment during the program period.

**Results:**

Between baseline and endline, zinc coverage increased from 2.5% to 22.4% in Gujarat and from 3.1% to 7.0% in UP; ORS coverage increased from 15.3% to 39.6% in Gujarat but did not change in UP. In comparison to baseline, children with diarrhea in the two–weeks preceding the endline survey had higher odds of receiving zinc treatment in both Gujarat (odds ratio, OR = 11.2; 95% confidence interval (CI) 6.4–19.3) and UP (OR = 2.4; 95% CI 1.4–3.9), but the odds of receiving ORS only increased in Gujarat (OR = 3.6; 95% CI 2.7–4.8; UP OR = 0.9; 95% CI 0.7–1.2). Seeking care outside the home, especially from a public sector source, was associated with higher odds of receiving ORS and zinc.

**Conclusions:**

During the duration of the DAZT program, there were modest improvements in the treatment of diarrhea among young children. Future programs should build upon and accelerate this trend with continued investment in public and private sector provider training and supply chain sustainability, in addition to targeted caregiver demand generation activities.

Despite absolute reductions in the global number of diarrhea–attributable deaths among children under–five over the past decade, diarrhea remains a leading cause of mortality in this age group [1]. In 2013, diarrhea caused an estimated 578 000 of the total 6.3 million under–five deaths [1]. In India, the number of under–five deaths attributable to diarrhea has decreased from 354 000 in 2000 to 140 000 in 2013 but continues to exceed that of any other country in the world [1].

Diarrhea is also responsible for significant morbidity among children in low– and middle–income countries worldwide. There were an estimated 1.731 billion episodes of diarrhea among children under–five in 2010, approximately 98% of which were mild or moderate [2]. Repeat bouts of less severe episodes that do not progress to death can result in long–term sequelae, such as poor nutritional status, stunting and subsequent decreases in cognitive function [2-4]. In India, this risk is substantial with children aged 0–5, 6–11, 12–23 and 24–59 months experiencing an average of 2.5, 3.8, 3.1 and 2.0 diarrheal episodes per year, respectively [5].

The diarrhea treatment guidelines supported by the Government of India and the Indian Academy of Pediatrics are in accordance with the WHO/UNICEF guidelines that include reduced osmolarity oral rehydration salts (ORS) and 14 days of zinc supplementation (20 mg of zinc/d for children ≥6 months and 10 mg of zinc/d for children 2–5 months of age) [6,7]. However, despite national recommendations, the most recent National Family Health Survey (NFHS) reported ORS coverage of 26% and zinc coverage of less than 1% [8]. Focused scale–up efforts are therefore warranted but have been slow to roll–out in many states. In response to this need, the Bill and Melinda Gates Foundation funded the Diarrhea Alleviation through Zinc and ORS Treatment (DAZT) program in Gujarat from 2011–2013 and in Uttar Pradesh (UP) from 2011–2014.

The main objective of the DAZT program was to scale–up adequate treatment of diarrhea among children under–five through public and private sector channels in selected districts. Micronutrient Initiative (MI) and FHI360 were tasked with carrying out project activities in the public and private sectors, respectively. The Johns Hopkins Bloomberg School of Public Health Institute for International Programs (JHSPH IIP) and in–country partner, the Society for Applied Studies (SAS), were responsible for conducting a large–scale external effectiveness evaluation to assess changes in diarrhea careseeking and ORS and zinc coverage over the project period. In this paper, we present the results of household coverage surveys conducted before and after program implementation in both states as part of this effectiveness evaluation. The baseline coverage surveys were carried out in 2011 in both states and the endline surveys were conducted in 2013 in Gujarat and in 2014 in UP.

## METHODS

### Evaluation context: study population

**Figure 1:**
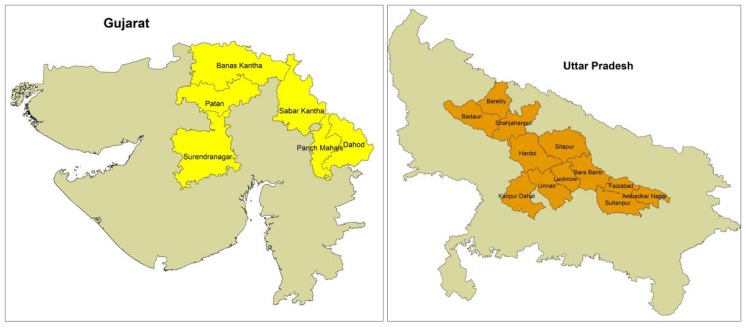
Map of the DAZT program districts in Gujarat and Uttar Pradesh, India. 6 program districts in Gujarat (*Banas Kantha, Dohad, Panch Mahals, Patan, Sabar Kantha Surendranagar*) and 12 program districts in UP (*Ambedkar Nagar, Bara Banki, Bareilly, Budaun, Faizabad, Hardoi, Kanpur Dehat, Lucknow, Shahjahanpur, Sitapur, Sultanpur, Unnao*). The map was generated using ArcGIS software and DIVA–GIS shapefiles [11,12].

Gujarat and UP are representative of the various sub–national child health and economic development contexts existent within India. Of the 29 Indian states, Gujarat has the third highest GDP per capita, whereas UP ranks 26th [9]. According to the 2011 census, Gujarat’s population of 60 million is the 9th largest in India but is small in comparison to that of UP, which is the most highly populated state with over 199 million inhabitants [10]. The DAZT program was implemented in 6 districts in Gujarat (**Figure 1**) with a total population of 13.2 million and approximately 2.1 million children under–five [10]. In UP, the program was implemented in 12 districts **(****Figure 1****)** with total and under–five populations of approximately 41.1 million and 6.3 million, respectively [10].

In 2007, 27% of India’s under–five deaths occurred in UP compared to 5% in Gujarat [13]. Although lower in Gujarat than in UP, Gujarat has the 6th highest absolute number of under–five deaths in India, outranking other states with poorer economic development and larger populations [13]. Diarrhea is a leading cause of under–five mortality in both Gujarat and UP. Prior to implementation of the DAZT program, the most recently available ORS coverage estimates of 26.3% in Gujarat and 12.5% in UP, highlighted the substantial need for focused scale–up in both states [14,15].

### Evaluation context: program design

Detailed descriptions of the specific public and private sector activities of the DAZT program have been published elsewhere [16,17]. In brief, the public sector program focused on provision of training to facility–based medical officers and auxiliary nurse midwives (ANMs) and to community–level Accredited Social Health Activists (ASHAs) and Anganwadi workers (AWWs). The training sessions covered overall diarrhea prevention and management with emphasis on ORS and zinc treatment. The public sector program also addressed ORS and zinc supply shortages by securing an initial seed supply of diarrhea treatment kits (DTKs) consisting of two ORS sachets and 14 zinc tablets. These DTKs were distributed to public sector facilities in the interim period before the state and district governments assumed responsibility for supply chain management.

In the private sector, the program engaged both formally qualified doctors and informal providers. The latter cadre of informal private sector providers often lack government–approved degrees and/or licences and consequently operate underground; however, they provide the bulk of diarrhea treatment in many remote rural villages. In order to reach formal and informal private providers (PPs), the program implementers enlisted local non–governmental organizations (NGO) and pharmaceutical companies to visit PPs at their places of work. During these visits, the NGO and pharmaceutical representatives showed PPs videos about adequate diarrhea treatment and solicited the sale of zinc syrups and/or tablets. Representatives made repeat zinc solicitation visits to PPs; the frequency of visits was based on the provider’s patient load and thus zinc–prescribing potential.

### Evaluation study design

We conducted an external evaluation of the DAZT program with a prospective, quasi–experimental, pre–post design. The main evaluation activities centered on cross–sectional household surveys at baseline and endline to assess changes in diarrhea careseeking and treatment among children aged 2–59 months in intervention districts. The target population excluded infants <2 months because zinc is not advised for this age group according to the Government of India guidelines. Baseline data were collected from March–June 2011 in both states. Due to government elections that resulted in unforeseen project delays in UP, the timing of endline data collection differed by state; the endline was conducted from September–November 2013 in Gujarat and from August–October 2014 in UP.

### Sample size calculations

Sample size calculations were designed to ensure adequate power to detect ORS rather than zinc coverage, since pre–DAZT zinc coverage was close to 0% in both states. For the baseline surveys, we calculated the state–specific sample sizes required for a precision estimate of ORS coverage ±7% at the alpha = 5% level, assuming coverage of 26.3% in Gujarat and 12.5% in UP as reported by the most recently conducted national survey [14,15]. At endline, we calculated the state–specific sample sizes required to detect a 10% change in ORS coverage from the level observed at baseline with 80% power at the alpha = 5% level. For both surveys, the resulting sample sizes were inflated to ensure adequate power among the two poorest wealth quintiles and to account for within–village correlation and an anticipated refusal rate of 15%. The Gujarat calculations yielded minimum sample size requirements of 375 and 398 children with diarrhea in the two–weeks preceding the survey at baseline and endline, respectively. The UP calculations yielded a minimum baseline sample size of 350 and a minimum endline sample size of 707 children with diarrhea. All sample size calculations were conducted using Stata statistical software [18,19].

### Sampling design and survey procedures

For each survey, we applied two–week diarrhea prevalence to the required sample sizes in order to estimate the number of households required to achieve the necessary number of children with diarrhea in the preceding two–weeks. The respective number of households required at baseline and endline were 4200 and 5080 in Gujarat and 3889 and 7853 in UP. To ensure equal representativeness across the DAZT districts in each state, we divided the number of households evenly across the 6 districts in Gujarat and the 12 districts in UP. For each district, we employed a probability proportional to size (PPS) sampling strategy to randomly select villages on the basis of the most recently available village population census [10].

In each randomly selected village, the trained data collection team mapped and divided the area into clusters of *mahallahs* (ie, neighborhoods/blocks). The team started at a central point from within each cluster and employed the right hand rule to select households to screen for study inclusion. The screening process entailed inquiring as to whether a child aged 2–59 months resided within the household and, if so, whether the child’s primary caregiver was available at the time of the visit; in multi–family households with more than one eligible caregiver, only one was selected for inclusion. The teams visited households until either a maximum of 50 caregivers of children 2–59 months of age had been enrolled or all households in the village had been visited. The team continued to visit randomly selected villages sequentially until the required number of households was met in each district.

Trained interviewers obtained informed consent from each caregiver prior to administering the survey. Interviewers read the consent document aloud and caregivers provided a signature or fingerprint (if illiterate) to indicate willingness to participate. The interviewers subsequently administered the survey to consenting caregivers. The survey included questions on household characteristics, diarrhea management knowledge and typical diarrhea careseeking and treatment practices. Extended questions on careseeking and treatment were administered to caregivers of children who had experienced a diarrheal episode in the two–weeks prior to the survey; diarrhea was defined as the passage of at least 3 loose or watery stools in a 24–hour period. If the caregiver was responsible for more than one child aged 2–59 months, she was asked to base survey responses on the youngest child in that age range.

The consent and survey procedures were conducted in Gujarati in Gujarat and in Hindi in UP. Translated forms were back–translated into English to verify the quality of translation, as well as consistency across the Gujarati and Hindi versions.

### Statistical analyses

All statistical analyses were conducted using Stata 12.0 statistical software [19]. We conducted exploratory data analyses on household characteristics, caregiver diarrhea management knowledge, and diarrhea careseeking and treatment practices for both typical diarrheal episodes and episodes experienced in the two–weeks prior to the survey. For each state, we stratified responses by the experience of diarrhea in the preceding two–weeks and conducted t–tests and χ^2^ tests to assess the equivalence of survey responses between the baseline and endline populations.

To address the main evaluation question of whether ORS and zinc treatment of children with diarrhea in the two–weeks preceding the survey increased from baseline to endline, we constructed state–specific logistic regression models to compute crude and adjusted odds ratios (OR) and 95% confidence intervals (CI) for the receipt of ORS/zinc by study phase (ie, endline vs baseline). We employed generalized estimating equations (GEE) with the logit link function and an independent correlation structure to adjust for village–level clustering [20]. We identified potential confounders for inclusion in multivariable models on the basis of *a priori* knowledge and bivariate analyses showing an association with both study phase and the receipt of ORS/zinc. The final multivariable models included indicators of child’s sex, child’s age >1 year and caregiver’s education ≥1 year of schooling. Additionally, the ORS models included indicators of receipt of zinc and report of *pani ki kami*, a local term for dehydration; and the zinc models included an indicator of receipt of ORS and a continuous variable for maximum stool frequency in stools per day.

All models also included a categorical variable for careseeking, which was defined as no careseeking, private sector careseeking or public sector careseeking. We conducted a sensitivity analysis to assess how to best categorize the careseeking variable for the small proportion of caregivers who utilized both private and public sector sources. The results showed no statistically significant difference in the adjusted odds ratios between models allocating this small population into its own public/private sector careseeking category as compared to the private sector or the public sector categories. Given the comparable results, we opted to simplify the model by not adding an additional public/private sector category. To help stabilize relatively small frequencies of public sector careseekers, we opted to include caregivers who sought care through both sectors in the public sector careseeking category.

There were no missing values for key dependent and explanatory variables. We tested all models for interaction between the study phase and careseeking variables. For final models, we confirmed the adequacy of fit using the Hosmer–Lemeshow test of goodness–of–fit [21].

## RESULTS

### Characteristics of caregivers, children and households

**Figure 2:**
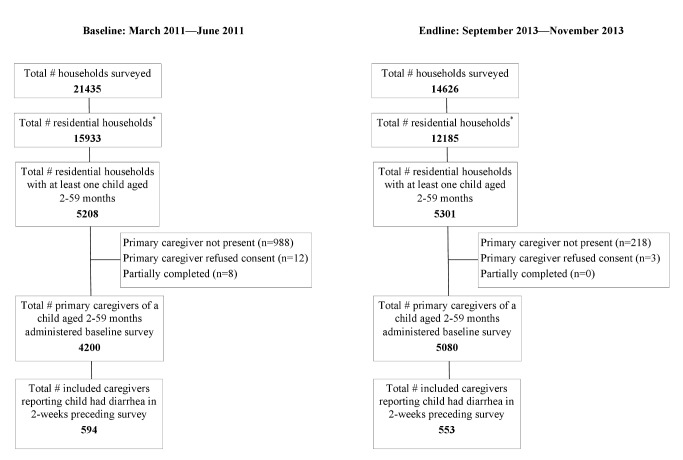
Survey profiles of the baseline and endline household surveys in Gujarat.

**Figure 3:**
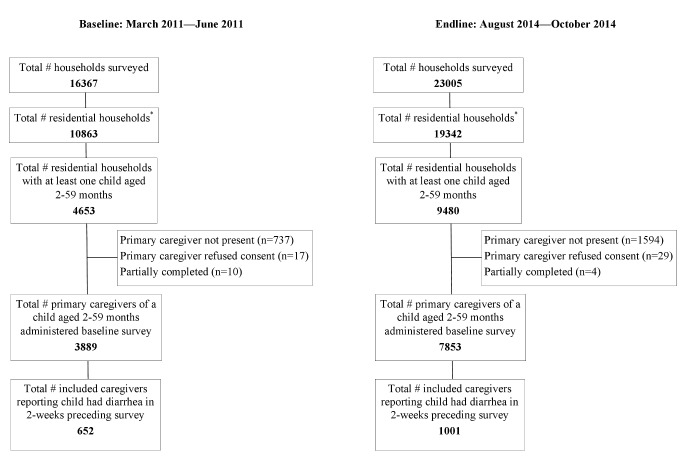
Survey profiles of the baseline and endline household surveys in Uttar Pradesh.

We collected baseline and endline data from 4200 and 5080 caregivers in Gujarat and from 3889 and 7853 caregivers in UP, respectively (**Figure 2** and **Figure 3**). In both states, the two–week diarrhea prevalence was higher at baseline than endline (Gujarat: 14.1% vs 10.9%, *P* < 0.001; UP: 16.8% vs 12.7%, *P* < 0.001). Characteristics of the caregiver, child and household were generally similar between baseline and endline in both states (**Table 1**). On average, caregivers at endline reported approximately one additional year of schooling compared to those at baseline in both states (*P* < 0.001). In both states, the mean age and the ratio of male–to–female children were statistically significantly equivalent comparing baseline to endline. In Gujarat, the proportion of households with purified drinking water was high for both surveys but slightly fewer households had access at endline (76.3%) than baseline (81.5%; *P* < 0.001). We observed the same trend in UP; although, the proportion of households reporting purified drinking water was substantially lower in the state (2.3% at baseline vs 0.8% at endline; *P* < 0.001). A larger proportion of households had access to a toilet facility at endline compared to baseline in both Gujarat (26.3% vs 21.6%, *P* < 0.001) and UP (26.6% vs 18.2%, *P* < 0.001). Additional characteristics of the study population are presented in **Table 1**.

**Table 1:** Characteristics of the primary caregiver, index child and household at baseline and endline, stratified by whether diarrhea was experienced by the index child in the two–weeks preceding the survey

Characteristics	Gujarat						Uttar Pradesh					
	No diarrhea in the last two–weeks (number, %)			Diarrhea in the last two–weeks (number, %)			No diarrhea in the last two–weeks (number, %)			Diarrhea in the last two–weeks (number, %)		
	Baseline (N = 3606)	Endline (N = 4527)	*P*–value*	Baseline (N = 594)	Endline (N = 553)	*P*–value*	Baseline (N = 3237)	Endline (N = 6852)	*P*–value*	Baseline (N = 652)	Endline (N = 1001)	*P*–value*
**Primary caregiver:†**												
Education in years of schooling:												
–Mean (SD)	3.9 (4.4)	4.6 (4.5)	<0.001‡	3.5 (4.2)	4.3 (4.3)	0.001‡	3.0 (4.4)	4.4 (5.0)	<0.001‡	2.7 (4.1)	3.8 (4.6)	<0.001‡
–Median (range)	2 (0, 22)	5 (0, 18)		0 (0, 17)	4 (0, 15)		0 (0, 19)	2 (0,19)		0 (0, 18)	0 (0, 17)	
Never attended school	1726 (47.9)	1848 (40.8)	<0.001†	314 (52.9)	228 (41.2)	<0.001‡	2000 (61.8)	3365 (49.1)	<0.001‡	416 (63.8)	520 (52.0)	<0.001‡
Mean age in years (SD)	27.1 (5.4)	26.9 (4.7)	0.075	26.4 (4.9)	26.6 (4.4)	0.471	28.6 (6.0)	28.2 (5.4)	<0.001‡	28.0 (5.5)	27.5 (4.9)	0.052
**Index child:†**												
Male	1982 (55.0)	2562 (56.6)	0.141	304 (51.2)	297 (53.7)	0.392	1723 (53.2)	3710 (54.1)	0.389	336 (51.5)	536 (53.6)	0.423
Mean age of child in months (SD)	25.5 (16.0)	24.4 (15.0)	0.001‡	16.7 (12.6)	20.3 (14.1)	<0.001‡	26.0 (16.0)	26.2 (15.7)	0.445	18.6 (13.6)	18.8 (13.5)	0.763
**Household:**												
Father’s years of schooling: Mean (SD)	7.1 (5.6)	7.7 (4.4)	<0.001‡	6.8 (4.4)	7.5 (4.0)	0.005‡	6.1 (4.9)	6.9 (5.0)	<0.001‡	5.9 (4.7)	6.3 (4.8)	0.118
Father’s years of schooling: Median (Range)	8 (0, 20)	9 (0, 20)		7 (0, 19)	8 (0, 21)		7 (0, 20)	8 (0, 22)		7 (0, 18)	8 (0, 19)	
Purified drinking water	2954 (81.9)	3475 (76.8)	<0.001‡	467 (78.6)	403 (72.9)	0.023‡	77 (2.4)	54 (0.8)	<0.001‡	11 (1.7)	6 (0.6)	0.032‡
Water on premises or <30 min to source	3119 (86.5)	3966 (87.6)	0.137	522 (87.9)	487 (88.1)	0.929	3209 (99.1)	6800 (99.2)	0.575	648 (99.4)	994 (99.3)	0.834
Household access to toilet facility§	809 (22.4)	1224 (27.0)	<0.001‡	97 (16.3)	114 (20.6)	0.061	599 (18.5)	1865 (27.2)	<0.001‡	107 (16.4)	222 (22.2)	0.004‡
BPL (below poverty line) card	1431 (39.7)	2164 (47.8)	<0.001‡	254 (42.8)	277 (50.1)	0.013	891 (27.5)	1547 (22.6)	<0.001‡	198 (30.4)	215 (21.5)	<0.001‡
Religion of father/ head of the household:			0.205			0.546			0.001‡			0.494
–Hindu	3443 (95.2)	4331 (95.7)		569 (95.8)	537 (97.1)		2865 (88.5)	5908 (86.2)		555 (85.1)	862 (86.1)	
–Muslim	164 (4.6)	178 (3.9)		22 (3.7)	14 (2.5)		369 (11.4)	942 (13.8)		97 (14.9)	137 (13.7)	
–Other	9 (0.3)	18 (0.4)		3 (0.5)	2 (0.4)		3 (0.1)	2 (0.03)		0	2 (0.2)	
Ethnic group:			<0.001†			0.006			0.273			0.803
–Scheduled caste	434 (12.0)	754 (16.7)		79 (13.3)	110 (19.9)		1140 (35.2)	2510 (36.6)		235 (36.0)	377 (37.7)	
–Scheduled tribe	1088 (30.2)	1125 (24.9)		200 (33.7)	165 (29.8)		7 (0.2)	7 (0.1)		0	0	
–Other backward caste	1495 (41.5)	2030 (44.8)		238 (40.1)	227 (41.1)		1546 (47.8)	3211 (46.9)		317 (48.6)	475 (47.5)	
–Other	589 (16.3)	618 (13.7)		77 (13.0)	51 (9.2)		544 (16.8)	1124 (16.4)		100 (15.3)	149 (14.9)	

*P–values were generated comparing baseline and endline values in Stata 12.0 using t–tests of equivalence for continuous variables and χ^2^ tests (or Fischer's exact tests in the case of low cell frequencies) for binary and categorical variables [19].

†Primary caregiver was the survey respondent. The index child was the respondent's youngest child in the 2–59 mo age group on whom all survey responses were based.

‡Statistically significant at the *P* = 0.05 level.

§Includes households with own or shared access to one of the following facilities: flush; flush–to–piped–sewer; septic tank; pit latrine with or without slab/open pit. Households without toilet facilities reported open defecation.

### Caregiver knowledge of diarrhea careseeking and treatment

Caregiver knowledge of appropriate sources of care for a child with diarrhea improved from baseline to endline in both states (**Table 2**). Awareness of private sector sources was high at baseline and experienced a moderate increase at endline (Gujarat: 92.6% vs 94.6%, *P* < 0.001; UP: 98.4% vs 99.7%, *P* < 0.001). In comparison to baseline, there was a large statistically significant increase in the proportion of caregivers who reported public sector sources as appropriate channels for diarrhea careseeking at endline in both states (Gujarat: 59.2% vs 89.1%, *P* < 0.001; UP: 25.8% vs 76.7%, *P* < 0.001); although, the absolute increase was more pronounced in UP (50.9%) than Gujarat (29.9%). In Gujarat, improved public sector awareness was largely driven by increased recognition of ASHAs (4.2% vs 44.2%, *P* < 0.001) and AWWs as appropriate sources of diarrhea treatment (15.5% vs 46.3%, *P* < 0.001); whereas in UP, the shift was primarily driven by increased recognition of primary health centers (PHCs) (25.5% vs 71.9%, *P* < 0.001) and, to a lesser degree, ASHAs (0.03% vs 17.9%, *P* < 0.001).

**Table 2:** Caregiver knowledge of diarrhea careseeking and treatment, stratified by whether diarrhea was experienced by the index child in the two–weeks preceding the survey

	Gujarat						Uttar Pradesh					
**No diarrhea in the last two weeks (No., %)**			**Diarrhea in the last two weeks** **(No., %)**			**No diarrhea in the last two weeks (No., %)**			**Diarrhea in the last two weeks (No., %)**		
**Baseline (N = 3606)**	**Endline (N = 4527)**	*P*–value*	**Baseline (N = 594)**	**Endline (N = 553)**	*P*–value*	**Baseline (N = 3237)**	**Endline (N = 6852)**	*P*–value*	**Baseline (N = 652)**	**Endline (N = 1001)**	*P*–value*
**Appropriate sources of care for a child with diarrhea**:†												
**Public sector source**	2123 (58.9)	4010 (88.6)	<0.001‡	363 (61.1)	515 (93.1)	<0.001‡	834 (25.8)	5229 (76.3)	<0.001‡	168 (25.8)	791 (79.0)	<0.001‡
–PHC/Government hospital	1837 (50.9)	3312 (73.2)	<0.001‡	315 (53.0)	418 (75.6)	<0.001‡	825 (25.5)	4898 (71.5)	<0.001‡	167 (25.6)	748 (74.7)	<0.001‡
–Auxiliary nurse midwife	257 (7.1)	508 (11.2)	<0.001‡	45 (7.6)	62 (11.2)	0.034‡	11 (0.3)	109 (1.6)	<0.001‡	1 (0.2)	18 (1.8)	0.002‡
–Accredited social health activist (ASHA)	159 (4.4)	2010 (44.4)	<0.001‡	19 (3.2)	234 (42.3)	<0.001‡	1 (0.03)	1248 (18.2)	<0.001‡	0	157 (15.7)	<0.001‡
–Anganwadi worker (AWW)	555 (15.4)	2063 (45.6)	<0.001‡	94 (15.8)	287 (51.9)	<0.001‡	2 (0.06)	322 (4.7)	<0.001‡	0	42 (4.2)	<0.001‡
**Private sector source**	3340 (92.9)	4287 (94.7)	<0.001‡	551 (92.8)	519 (93.9)	0.461	3181 (98.3)	6828 (99.7)	<0.001‡	644 (98.8)	999 (99.8)	<0.001‡
–Private provider	2784 (77.2)	3785 (83.6)	<0.001‡	461 (77.6)	471 (85.2)	0.001‡	3136 (96.9)	6730 (98.2)	<0.001‡	631 (96.8)	983 (98.2)	0.063
–Private hospital/nursing home	1539 (42.7)	1059 (23.4)	<0.001‡	224 (37.7)	129 (23.3)	0.001‡	323 (10.0)	1878 (27.4)	<0.001‡	76 (11.7)	283 (28.3)	<0.001‡
–Chemist	138 (3.8)	1494 (33.0)	<0.001‡	14 (2.4)	174 (31.5)	0.001‡	22 (0.7)	2515 (36.7)	<0.001‡	3 (0.5)	437 (43.7)	<0.001‡
–Traditional healer	18 (0.5)	147 (3.3)	<0.001‡	5 (0.8)	22 (4.0)	0.001‡	0	81 (1.2)	<0.001‡	0	21 (2.1)	<0.001‡
–Charitable hospital/NGO/Trust	28 (0.8)	32 (0.7)	0.716	13 (2.2)	6 (1.1)	0.143	2 (0.06)	3 (0.04)	0.705	0	0	–
–Mobile clinic	44 (1.2)	64 (1.4)	0.449	4 (0.7)	8 (1.5)	0.198	19 (0.6)	195 (2.9)	<0.001‡	2 (0.3)	12 (1.2)	0.053
Aware of ORS for diarrhea treatment§	1942 (53.9)	3397 (75.0)	<0.001‡	315 (53.0)	466 (84.3)	<0.001‡	2685 (82.9)	5399 (78.8)	<0.001‡	561 (86.0)	787 (78.6)	<0.001‡
Aware of zinc for diarrhea treatment§	163 (4.5)	976 (21.6)	<0.001‡	26 (4.4)	200 (36.2)	<0.001‡	168 (5.2)	2022 (29.5)	<0.001‡	51 (7.8)	389 (38.9)	<0.001‡
–If yes, source of zinc awareness:†,#												
–Public sector source	39 (23.9)	769 (78.8)	<0.001‡	4 (15.4)	155 (77.5)	<0.001‡	19 (11.3)	739 (36.6)	<0.001‡	7 (13.7)	107 (27.5)	0.035‡
–Private sector source	136 (83.4)	540 (55.3)	<0.001‡	24 (92.3)	112 (56.0)	<0.001‡	137 (81.6)	1449 (71.7)	0.006‡	40 (78.4)	303 (77.9)	0.930
–Media (announcement, radio, poster/wall painting, pamphlet, television)	1 (0.6)	84 (8.6)	<0.001‡	0	11 (5.5)	0.220	5 (3.0)	92 (4.6)	0.341	5 (9.8)	13 (3.3)	0.029‡
–Neighbor/relative	1 (0.6)	47 (4.8)	<0.001‡	0	11 (5.5)	0.220	25 (14.9)	187 (19.1)	0.175	3 (5.9)	83 (21.3)	<0.009‡

NGO – non–governmental organizations

*P–values were generated comparing baseline and endline values in Stata 12.0 using t–tests of equivalence for continuous variables and χ^2^ tests (or Fischer's exact tests in the case of low cell frequencies) for binary and categorical variables [19].

†Respondents could supply more than one answer; column percentage totals may exceed 100%.

‡Statistically significant at the *P* = 0.05 level.

§Respondents were considered aware of ORS/zinc for diarrhea treatment if they reported having seen or heard the products prior to the survey and responded (unprompted) that ORS/zinc were used for diarrhea treatment.

#Percentages based on denominator of total aware of zinc for diarrhea treatment.

We observed a statistically significant increase in ORS awareness from 53.7% at baseline to 76.0% at endline in Gujarat (*P* < 0.001) (**Table 2**). In UP, ORS awareness decreased by an absolute difference of 4.7% (*P* < 0.001) comparing baseline to endline. There was a statistically significant increase in the proportion of caregivers who had seen or heard of zinc and, without prompting, recognized it as a treatment for diarrhea in both Gujarat (4.5% vs 23.2%; *P* < 0.001) and UP (5.6% vs 30.7%; *P* < 0.001). In both states, higher zinc awareness at endline was attributed to increased report of public sector sources of information on zinc as a treatment for diarrhea. In **Table 2**, we report additional data on caregiver knowledge of diarrhea careseeking and treatment stratified by report of diarrhea in the two–weeks prior to the survey; the trends were similar comparing caregivers with and without a recent diarrheal episode.

### Careseeking and treatment of recent diarrheal episodes

The reported characteristics of diarrheal episodes occurring in the two–weeks preceding the survey were generally similar at baseline and endline in both states (**Table 3**). In Gujarat, *pani ki kami* (a local term for dehydration), lethargy/irritability and sunken eyes were less frequently reported at endline (*P* < 0.001). In UP, vomiting and sunken eyes were less common episode characteristics at endline (*P* < 0.001), and mean maximum stool frequency decreased by 1.3 stools/d (*P* < 0.001).

**Table 3:** Careseeking and treatment of diarrheal episodes occurring in the two–weeks preceding the survey

	Gujarat (No., %)			Uttar Pradesh (No., %)		
**Baseline (N = 594)**	**Endline (N = 553)**	*P*–value*	**Baseline (N = 652)**	**Endline (N = 1001)**	*P*–value*
**Episode characteristics:**						
Blood in stools	44 (7.4)	34 (6.2)	0.397	108 (16.6)	166 (16.6)	0.985
Fever	370 (62.3)	318 (57.5)	0.098	543 (83.3)	859 (85.9)	0.147
Vomiting	218 (36.7)	199 (36.0)	0.801	377 (57.8)	377 (37.7)	<0.001**
*Pani ki kami* (local term for dehydration)	268 (45.1)	167 (30.2)	<0.001†	344 (52.8)	573 (57.3)	0.070
Lethargy/irritability	382 (64.3)	245 (44.3)	<0.001†	487 (74.7)	780 (78.0)	0.120
Sunken eyes	156 (26.3)	48 (8.7)	<0.001†	271 (41.6)	300 (30.0)	<0.001**
Maximum stool frequency (stools/d)						
Mean (SD)	4.8 (1.5)	4.8 (1.4)	0.835	6.6 (3.1)	5.3 (2.1)	<0.001**
Median (range)	4 (3, 13)	4 (3, 10)		6 (3, 30)	5 (3, 25)	
**Sought care outside the home**	398 (67.0)	412 (74.5)	0.005†	572 (87.7)	855 (85.4)	0.178
**If yes, source of careseeking:**‡,§						
Public sector source	79 (19.6)	155 (37.6)	<0.001†	25 (4.4)	78 (9.1)	<0.001†
–Primary health center/Government hospital	55 (13.8)	72 (17.5)	0.153	25 (4.4)	59 (6.9)	0.047†
–Auxiliary nurse midwife	4 (1.0)	8 (1.9)	0.270	0	1 (0.1)	0.413
–Accredited social health activist (ASHA)	2 (0.5)	37 (9.0)	<0.001†	0	16 (1.9)	0.001†
–Anganwadi Worker (AWW)	18 (4.5)	53 (12.9)	<0.001†	1 (0.2)	3 (0.4)	0.538
Private sector source	319 (80.2)	306 (74.3)	0.046†	532 (93.0)	773 (90.4)	0.086
–Private provider	227 (57.0)	264 (64.1)	0.040†	487 (85.1)	689 (80.6)	0.027†
–Private hospital/Nursing home	64 (16.1)	27 (6.6)	<0.001†	24 (4.2)	32 (3.7)	0.666
–Chemist	47 (11.8)	30 (7.3)	0.028†	28 (4.9)	77 (9.0)	0.004†
–Traditional healer#	4 (1.0)	5 (1.2)	0.777	3 (0.5)	11 (1.3)	0.152
–Charitable hospital/NGO/Trust	5 (1.3)	3 (0.7)	0.447	0	1 (0.1)	0.413
–Mobile clinic	1 (0.3)	1 (0.3)	0.981	1 (0.2)	4 (0.5)	0.359
–General store	26 (6.5)	9 (2.2)	0.003†	28 (4.9)	64 (7.5)	0.050
**Administered treatment:**						
Any treatment	418 (70.4)	429 (77.6)	0.006†	582 (89.3)	911 (91.0)	0.244
ORS¶	91 (15.3)	219 (39.6)	<0.001†	141 (21.6)	202 (20.2)	0.485
Zinc¶	15 (2.5)	124 (22.4)	<0.001†	20 (3.1)	70 (7.0)	<0.001†
ORS and zinc¶	3 (0.5)	102 (18.4)	<0.001†	8 (1.2)	33 (3.3)	0.008†
Antibiotics	95 (16.0)	177 (32.0)	<0.001†	184 (28.2)	308 (30.8)	0.259
Antidiarrheal	31 (5.2)	121 (21.9)	<0.001†	17 (2.6)	426 (42.6)	<0.001†
Syrup, unknown	132 (22.2)	51 (9.2)	<0.001†	127 (19.5)	113 (11.3)	<0.001†
Tablet, unknown	213 (35.9)	57 (10.3)	<0.001†	165 (25.3)	282 (28.2)	0.196
Powder, unknown	47 (7.9)	10 (1.8)	<0.001†	213 (32.7)	4 (0.4)	<0.001†
Injection	40 (6.7)	12 (2.2)	<0.001†	165 (25.3)	125 (12.5)	<0.001†
IV Fluids	5 (0.8)	8 (1.5)	0.334	13 (2.0)	2 (0.2)	<0.001†

ORS – oral rehydration salts, NGO – non–governmental organization, IV – intravenous

*P–values were generated comparing baseline and endline values in Stata 12.0 using t–tests of equivalence for continuous variables and χ^2^ tests (or Fischer's exact tests in the case of low cell frequencies) for binary and categorical variables (16).

†Statistically significant at the *P* = 0.05 level.

‡Respondents could supply more than one answer; column percentage totals may exceed 100%.

‡Percentages based on denominator of total who sought care outside the home.

#Includes Ayurvedic, Vaid, Homepathic, Hakim, Unani.

¶ORS category includes children who received ORS with or without zinc and vice versa. ORS and zinc category includes children who received both products.

**Figure 4:**
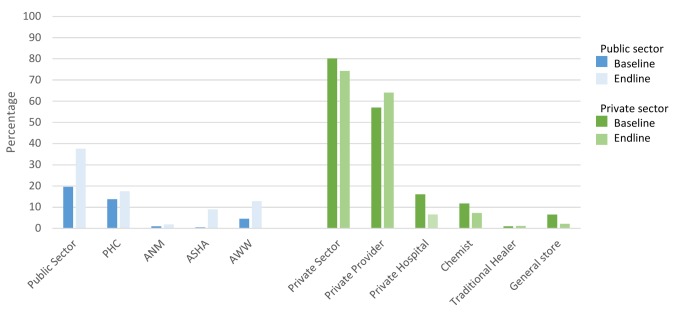
Sources of diarrhea careseeking at baseline and endline in Gujarat. Public sector includes: primary health centers, auxiliary nurse midwives, Accredited Social Health Activities and Anganwadi workers; private sector includes: private providers, private hospitals, chemists, traditional healers and general stores.

**Figure 5:**
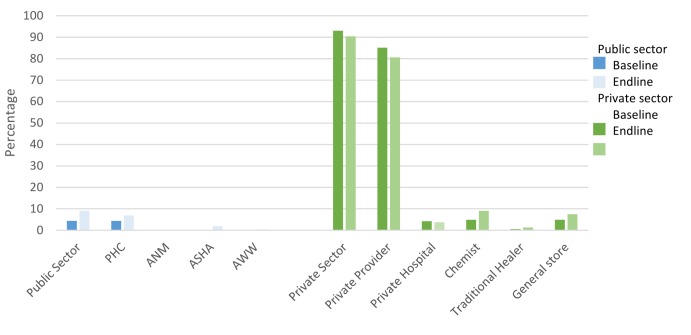
Sources of diarrhea careseeking at baseline and endline in Uttar Pradesh. Public sector includes: primary health centers, auxiliary nurse midwives, Accredited Social Health Activities and Anganwadi workers; private sector includes: private providers, private hospitals, chemists, traditional healers and general stores.

The proportion of caregivers who sought care outside the home for their child’s diarrhea increased slightly from baseline to endline in Gujarat (67.0% vs 74.5%, *P* = 0.005) but did not change in UP (87.7% vs 85.4%, *P* = 0.178) (**Table 3**). There was a statistically significant increase in public sector careseeking in both states, although the trend was more pronounced in Gujarat (19.6% vs 37.6%, *P* < 0.001; **Figure 4**) compared to UP (4.4% vs 9.1%, *P* < 0.001; **Figure 5**). There was a borderline statistically significant decrease in private sector careseeking in Gujarat (80.2% vs 74.3%, *P* = 0.046) but no change in UP (93.0% vs 90.4%, *P* = 0.086). The shift in careseeking was characterized by increased attendance at ASHAs and AWWs in Gujarat and ASHAs and PHCs in UP. In Gujarat, the overall decrease in private sector utilization was driven by reduced use of private hospitals/nursing homes, chemists and general stores, despite a slight increase in use of private providers.

In Gujarat, the proportion of diarrheal episodes treated with ORS increased from 15.3% at baseline to 39.6% at endline (*P* < 0.001) (**Table 3**). In UP, there was no difference in ORS coverage at endline (20.2%) compared to baseline (21.6%, *P* = 0.485). Zinc treatment was statistically significantly higher at endline than baseline in both Gujarat (2.5% vs 22.4%, *P* < 0.001) and UP (3.1% vs 7.0%, *P* < 0.001). The same trend was observed in the number of episodes treated with both ORS and zinc (0.5% vs 18.4%, *P* < 0.001 in Gujarat and 1.2% vs 3.3%, *P* < 0.001 in UP).

There was a statistically significant increase in the proportion of children treated with antidiarrheals from baseline to endline in UP (5.2% vs 21.9%, *P* < 0.001) and Gujarat (2.6% vs 42.6%, *P* < 0.001; **Table 3**). This shift was driven by comparable increases in treatment with antidiarrheals through both the public and private sectors (data not shown). Among children who sought care in Gujarat, the proportion treated with antidiarrheals increased from 7.7% to 21.3% in the public sector (*P* < 0.001) and from 8.5% to 32.7% in the private sector (*P* < 0.001); in UP, this figure increased from 4.0% to 48.7% in the public sector (*P* < 0.001) and from 2.4% to 43.7% in the private sector (*P* < 0.001).

Compared to baseline, the proportion of children administered antibiotics was higher at endline in Gujarat (16% vs 32%, *P* < 0.001) but not in UP (28.2% vs 30.8%, *P* = 0.259; **Table 3**). Unlike the trend in antidiarrheals, the increase in antibiotics observed in Gujarat was solely driven by a rise in the proportion of children receiving antibiotics through the private sector (ie, 25.1% at baseline vs 50.3% at endline), as receipt of antibiotics through the public sector did not change between baseline and endline (ie, 23.1% vs 27.1%) (data not shown). Additional data on careseeking and treatment are provided in **Table 3**.

### Factors associated with ORS treatment

In bivariate analysis of the odds of ORS treatment at endline compared to baseline, there was a statistically significant increase in Gujarat (OR = 3.6, 95% CI 2.7–4.8) and a non–statistically significant decrease in UP (OR = 0.90, 95% CI 0.7–1.2) (**Table 4**).

**Table 4:** Bivariate and multivariate Generalized Estimating Equations* analyses of the association between study phase and receipt of ORS and zinc treatment among children with diarrhea in the two–weeks preceding the survey

	GUJARAT				UTTAR PRADESH			
**OUTCOME** – **RECEIPT OF ORS:**								
	Unadjusted OR (95% CI)	*P*–value	Adjusted OR (95% CI)†	*P*–value	Unadjusted OR (95% CI)	*P*–value	Adjusted OR (95% CI)†	*P*–value
Phase of study								
– Endline	3.6 (2.7–4.8)	<0.001‡			0.9 (0.7–1.2)	0.485	0.8 (0.6–1.0)	0.058
– Baseline	1.0				1.0		1.0	
Careseeking:								
– Public Sector	53.6 (28.8–99.5)	<0.001‡			10.8 (5.5–21.5)	<0.001‡	7.8 (3.9–15.7)	<0.001‡
– Private Sector	7.8 (4.4–14.1)	<0.001‡			4.6 (2.6–8.1)	<0.001‡	3.9 (2.2–6.9)	<0.001‡
– No careseeking	1.0				1.0		1.0	
Endline vs Baseline:‡								
– Public Sector	7.2 (3.9–13.3)	<0.001‡	4.7 (2.5–9.0)	<0.001‡				
– Private Sector	2.1 (1.4–3.1)	<0.001‡	1.6 (1.1–2.4)	0.036‡				
–No careseeking	4.9 (1.3–18.2)	0.017‡	4.7 (1.3–17.5)	0.021‡				
Receipt of zinc	12.1 (8.0–18.3)	<0.001‡	4.3 (2.6–7.0)	<0.001‡	3.5 (2.3–5.4)	<0.001‡	2.7 (1.7–4.2)	<0.001‡
**OUTCOME – RECEIPT OF ZINC:**								
	Unadjusted OR (95% CI)	*P*–value	Adjusted OR (95% CI)†	*P*–value	Unadjusted OR (95% CI)	*P*–value	Adjusted OR (95% CI)†	*P*–value
**Phase of study:**								
– Endline	11.2 (6.4–19.3)	<0.001‡	7.3 (4.1–13.0)	<0.001‡	2.4 (1.4–3.9)	0.001‡	2.5 (1.5–4.4)	0.001‡
– Baseline			1.0		1.0		1.0	
**Careseeking:**								
– Public Sector	87.6 (21.3–360.9)	<0.001‡	26.5 (6.1–114.7)	<0.001‡	16.8 (4.8–58.3)	<0.001‡	9.5 (2.7–33.6)	0.001‡
– Private Sector	18.4 (4.5–75.7)	<0.001‡	12.2 (2.9–51.6)	0.001‡	4.0 (1.3–12.9)	0.019	3.1 (1.0–10.1)	0.059
– No careseeking	1.0		1.0		1.0		1.0	
Receipt of ORS	12.1 (8.0–18.3)	<0.001‡	4.3 (2.6–7.0)	<0.001‡	3.5 (2.3–5.4)	<0.001‡	2.7 (1.7–4.3)	<0.001‡

OR – odds ratio, CI – confidence interval, ORS – oral rehydration salts

*Generalized estimating equations (GEE) with the logit link function and an independent correlation structure used to generate semi–robust standard errors to adjust for multiple observations at the village–level in Stata 12.0 (16).

†All multivariable analyses adjusted for the above–listed variables and: sex of child; age of child >1 y; and maternal education ≥1 y of school. ORS model also adjusted for receipt of zinc and report of *pani ki kami* (local term for dehydration). Zinc model also adjusted for receipt of ORS and reported maximum stool frequency in stools/d.

‡Statistically significant at the *P* = 0.05 level.

§Statistically significant interaction between phase of the study and careseeking sector (*P* = 0.009).

In multivariable analysis, there was a statistically significant interaction between the study phase and careseeking variables in Gujarat (*P* = 0.009). The adjusted odds (aOR) of ORS treatment at endline compared to baseline were elevated by a factor of 4.7 (95% CI 1.3–17.5) among children with no careseeking, by 1.6 (95% CI 1.1–2.4) among those with private sector careseeking and by 4.7 (95% CI 2.5–9.0) among those with public sector careseeking (**Table 4**). Among children for whom no care was sought, the higher odds of ORS treatment at endline compared to baseline were attributable to an increase in the proportion of caregivers who reported having ORS product at home. The adjusted odds of ORS treatment were higher among children who also received zinc (aOR = 4.3, 95% CI 2.6–7.0).

In UP, there was no interaction between the study phase and careseeking variables in multivariable analysis. The adjusted OR of ORS treatment comparing endline to baseline was 0.8 (95% CI 0.6–1.0) (**Table 4**). Compared to no careseeking, the adjusted odds of ORS treatment were 3.9 (95% CI 2.2–6.9) times higher among those who sought care from a private sector source and 7.8 (95% CI: 3.9–15.7) times higher among those who sought public sector care. The adjusted odds of ORS treatment were elevated by a factor of 2.7 (95% CI 1.7–4.2) among zinc–treated children.

### Factors associated with zinc treatment

The unadjusted odds of zinc treatment were statistically significantly higher at endline compared to baseline in both Gujarat (OR = 11.2, 95% CI 6.4–19.3) and UP (OR = 2.4, 95% CI 1.4–3.9) (**Table 4**). In multivariable analysis, the effect of study phase was not modified by careseeking in either state.

In Gujarat, the adjusted OR comparing zinc treatment at endline to baseline was 7.3 (95% CI 4.1–13.0) (**Table 4**). The adjusted ORs comparing zinc treatment among children with private and public sector careseeking relative to no careseeking were 12.2 (95% CI 2.9–51.6) and 26.5 (95% CI 6.1–114.7), respectively. In UP, the adjusted odds of zinc treatment were 2.5 (95% CI 1.5–4.4) times higher at endline compared to baseline and were elevated among children with private sector (aOR = 3.1, 95% CI 1.0–10.1) and public sector careseeking (aOR = 9.5, 95% CI 2.7–33.6) relative to those who did not seek care. In both states, there was a statistically significant association between zinc treatment and receipt of ORS (*P* < 0.001).

## DISCUSSION

The external evaluation of the DAZT program showed that over the course of the program period, the odds of zinc treatment increased in both states, and the odds of ORS treatment increased in Gujarat but not UP. In both states, the odds of adequate treatment were higher among those who sought care outside the home, but the effect was greater in the public compared to the private sector. Between baseline and endline, zinc awareness and recognition of public sector providers as appropriate sources of diarrhea care increased in both Gujarat and UP, and ORS awareness increased in Gujarat. Among caregivers of children with diarrhea in the preceding two–weeks, public sector careseeking was higher at endline relative to baseline, but private sector careseeking remained high. There was a decrease in diarrhea prevalence from baseline to endline, but this shift was likely attributable to the timing of the surveys within different diarrhea seasons and not to the DAZT program.

The design of the prospective evaluation was quasi–experimental and thus our conclusions are based on pre–post comparisons between the DAZT districts at baseline and endline. The use of historical controls is not the gold standard in evaluation design, but state government plans to eventually scale–up ORS and zinc throughout all districts in Gujarat and UP during the project period precluded the use of non–DAZT districts as comparison areas. In order to reduce the bias introduced from a quasi–experimental design, we routinely monitored the DAZT districts and collected data on potential contextual factors. Through this documentation, we did not become aware of any overlap between the DAZT project and other diarrhea management or sanitation programs that may have influenced ORS and zinc use in the selected area. Still, we are unable to definitively attribute changes in coverage to the DAZT program due to the limitations of our study design.

Our findings show that over the course of the DAZT project, zinc coverage increased in both states and ORS coverage increased in Gujarat. However, the magnitude of the change was not as large as anticipated, with only 18.4% and 3.3% of diarrheal episodes treated with both ORS and zinc at endline in Gujarat and UP, respectively. The need for improved diarrhea treatment among children under–five in the project areas is therefore still evident. Nevertheless, increases in ORS and zinc awareness and shifts in the recognition and utilization of public sector channels for diarrhea careseeking are promising first steps in generating program impact. It should be noted that these changes occurred in the absence of caregiver demand generation activities. Given the DAZT program’s sole focus on provider–level activities, changes in caregiver knowledge and practices could only have resulted through word–of–mouth. In particular, the public sector approach, which operated on the theory that improving the quality of diarrhea treatment among public providers would lead to increases in diarrhea careseeking through public sector channels, depended on the message of improved care to naturally trickle into the community. Our results indicate that perceptions regarding the role of public sector providers in diarrhea treatment were beginning to evolve among caregivers of young children; however, we observed gaps between the awareness of the public sector as an appropriate source of treatment and the practice of public sector careseeking. Future programs should incorporate community–level behavior change communication to quickly disseminate messages regarding appropriate sources of diarrhea treatment and to maximize the impact of those messages on careseeking practices. Moreover, activities targeting caregivers should also focus on generating demand for ORS and zinc, in addition to increasing awareness.

The evaluation results highlight differences in diarrhea treatment by the sector through which care was sought. The odds of receiving ORS and zinc were higher among those who sought care through either sector compared to those who did not seek care outside the home, but the effect was greater in the public sector. There are vast differences between the public and private sector health systems that could have contributed to variations in program impact. The public sector program may have been better positioned to modify providers’ diarrhea treatment practices because government employees are easily identifiable and can be required to attend trainings. In comparison, the private sector program had to contend with a large population of informal providers who were difficult to locate and at liberty to reject visits from program representatives. Despite the challenges associated with altering the diarrhea treatment practices of private providers, we observed only a gradual shift towards public sector careseeking with the overwhelming majority of caregivers continuing to seek care through private sources at endline, and thus future programmatic investment in the private sector is necessary and worthwhile.

The results of the evaluation underscored differences in the magnitude of change between states. In Gujarat, we observed absolute increases of 24.3% in ORS coverage and 19.9% in zinc coverage. In comparison, ORS coverage did not change and the absolute increase in zinc coverage was only 3.9% in UP. Since the odds of receiving ORS and zinc were higher in the public sector, it is possible that poorer coverage in UP compared to Gujarat was at least in part attributable to the relatively smaller increase in public sector careseeking (ie, 4.7% in UP vs 18.0% in Gujarat). There were also differences in the breakdown of public sector careseeking across specific provider cadres; careseeking to ASHAs and AWWs experienced absolute increases of more than 8% in Gujarat compared to 1.9% and 0.2% in UP. Future public sector programs in UP should focus on increasing the uptake of diarrhea careseeking through community–level ASHAs and AWWs.

The findings of this evaluation are potentially limited by the biases associated with caregiver report and recall. To reduce the threat of recall bias, we limited the assessment of diarrhea careseeking and treatment to episodes occurring within two–weeks preceding the survey, which is the widely accepted standard for large surveys [22]. In addition, we employed several methodological techniques to improve and confirm caregiver recall of diarrheal treatments given to children during the two–week period. During the interview, caregivers were shown laminated photos of commonly available diarrhea treatment products in an attempt to prompt recall of what had been administered to the child. Interviewers also checked all available packaging and recorded the product details. We increased our efforts to identify packaging at endline as an added precaution against misidentification of ORS and zinc; if packaging was torn and the brand name illegible, the interviewers brought the remnants to local chemists for assistance determining the identity of the product. As a result of the enhanced methods at endline, unknown treatments largely decreased in both states. Though a fraction of products could not be identified despite the added measures taken at endline, these were unlikely to have been zinc since the zinc product or packaging for a 10 to 14–day regimen would have been available in the household for episodes occurring within two weeks of the survey.

We observed an increasing trend in antidiarrheals and antibiotics from baseline to endline that is likely an artefact of the additional treatment identification methods employed at endline. It is possible that the revised methods, which resulted in an overall lower percentage of unknown products, produced an apparent increase in products identified as antidiarrheals and antibiotics. However, it is also possible that through heightened attention to diarrhea management, providers were not only more likely to advise ORS and zinc but also other misguided treatments. Future programs should be aware of this risk and focus efforts on warning providers of the dangers of mistreatment with antibiotics and antidiarrheals. In addition, future evaluations should be designed to assess these nuances, as well as the diarrhea treatment preferences and expectations of both providers and caregivers.

The results of the external evaluation of the DAZT program draw attention to factors of importance for future diarrhea management programs in Gujarat and UP, as well as generalizable areas throughout India and South Asia. An important conclusion of this evaluation is that the absence of demand generation activities targeting the community was a major flaw. The addition of activities aimed at generating demand for ORS and zinc among caregivers of young children would have complemented public and private sector activities. Community–level activities could have accelerated uptake of careseeking through ASHAs and AWWs, since lack of awareness of their ability to treat diarrhea was the leading reason reported at endline by caregivers who had never utilized ASHAs or AWWs for diarrhea care. Lack of diarrhea treatment supplies was an additional reason cited by caregivers who had never sought diarrhea treatment from ASHAs, AWWs or PHCs, thus underscoring the important link between preventing public sector ORS and zinc stock–outs and building trust among caregivers in the community. The results of the private sector evaluation demonstrate the potential role of private providers in provision of ORS and zinc despite the challenging nature of reaching informal providers; future programs may benefit from a systematic census to better characterize this population and facilitate coverage of the full universe of informal providers. Finally, our results suggest that provision of ORS and zinc are complementary in that the odds of receiving ORS increased with receipt of zinc and vice versa. This finding highlights the importance of emphasizing both ORS and zinc in training providers in either sector. Thus, as future diarrhea management programs are designed with the goal of introducing zinc, implementers must ensure that ORS is also given focus.
